# Supplementary material for: IL17F Expression as an Early Sign of Oxidative Stress-Induced Cytotoxicity/Apoptosis
Source: Genes (Basel). 2022 Sep 27;13(10):1739. doi: 10.3390/genes13101739 (PMC9602038; doi:10.3390/genes13101739)
Supplement: Supplementary file 1 [file genes-13-01739-s001.zip › genes-1870870-supplementary.pdf]

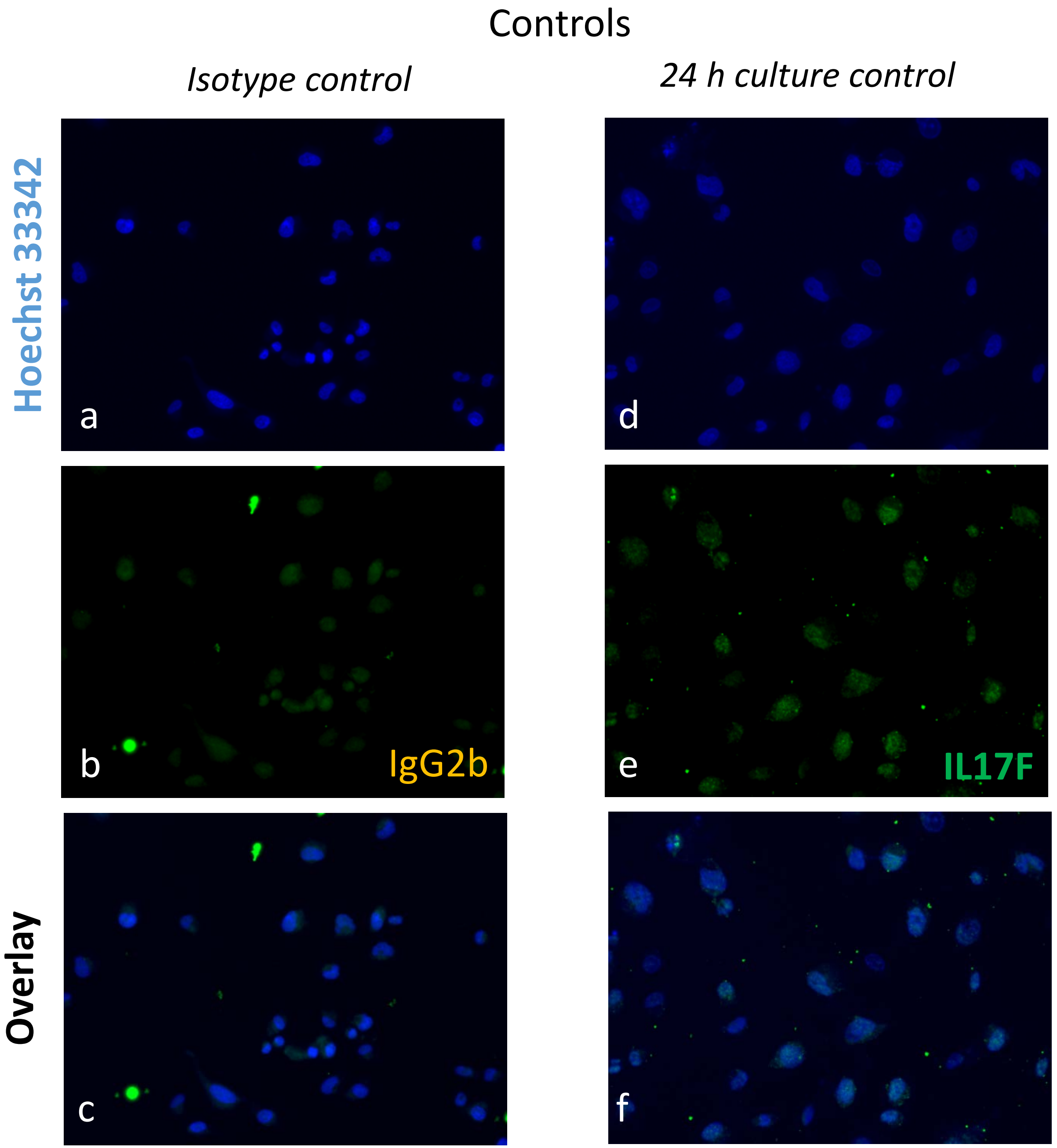

Supplementary Figure S1

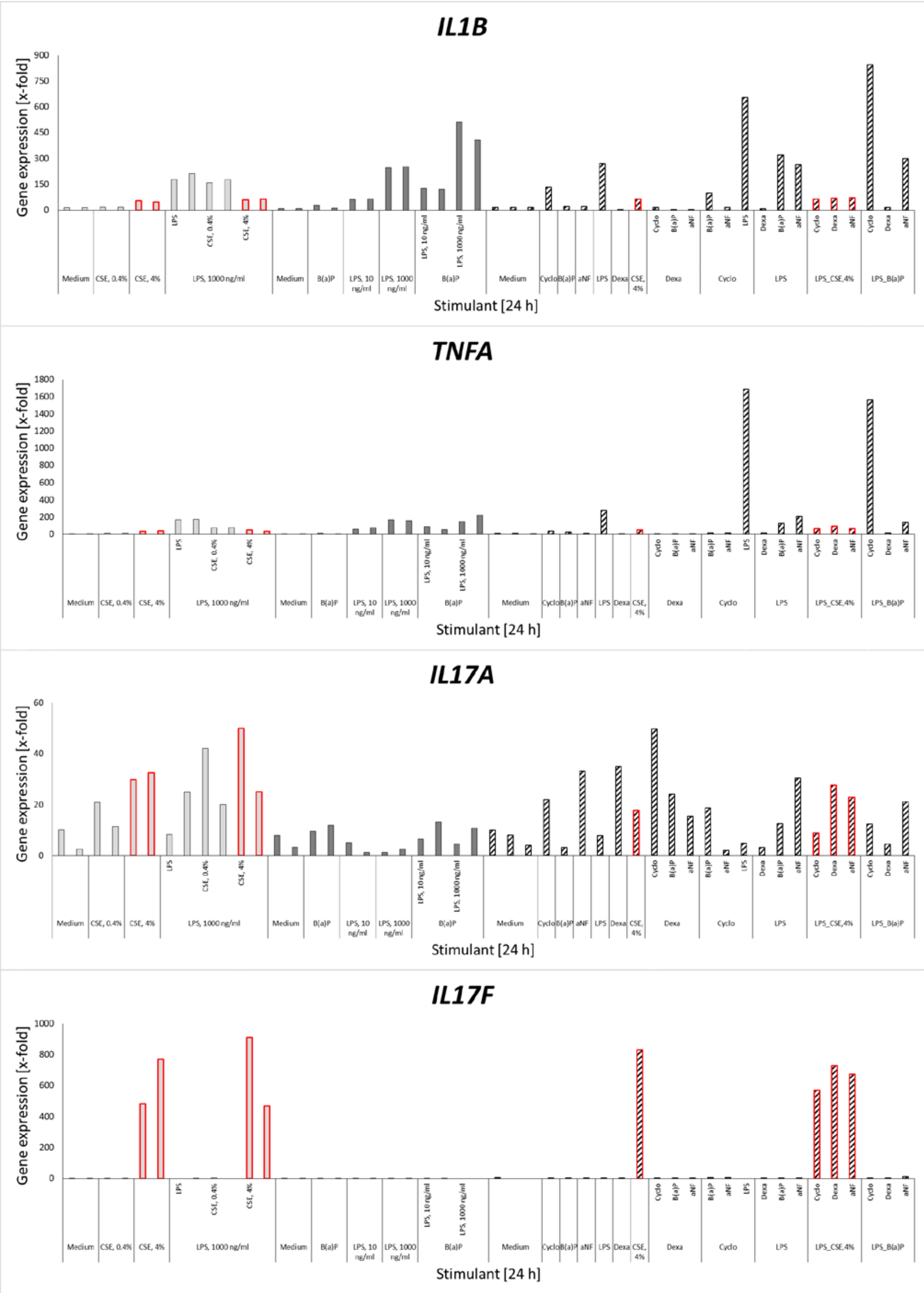

Stimulants of 3 independent exposure experiments (, ,):

Cyclo, cycloheximide *(translation inhibitor)*
Dexa, dexamethasone *(immunosuppressive glucocorticoid)*
aNF, alpha-naphthoflavone *(AhR-antagonist)*
B(a)P, benz(a)pyrene *(AhR-agonist)*
LPS, lipopolysaccharide *(TLR4-agonist)*
CSE, aqueous cigarette smoking extract

Supplementary Figure S2

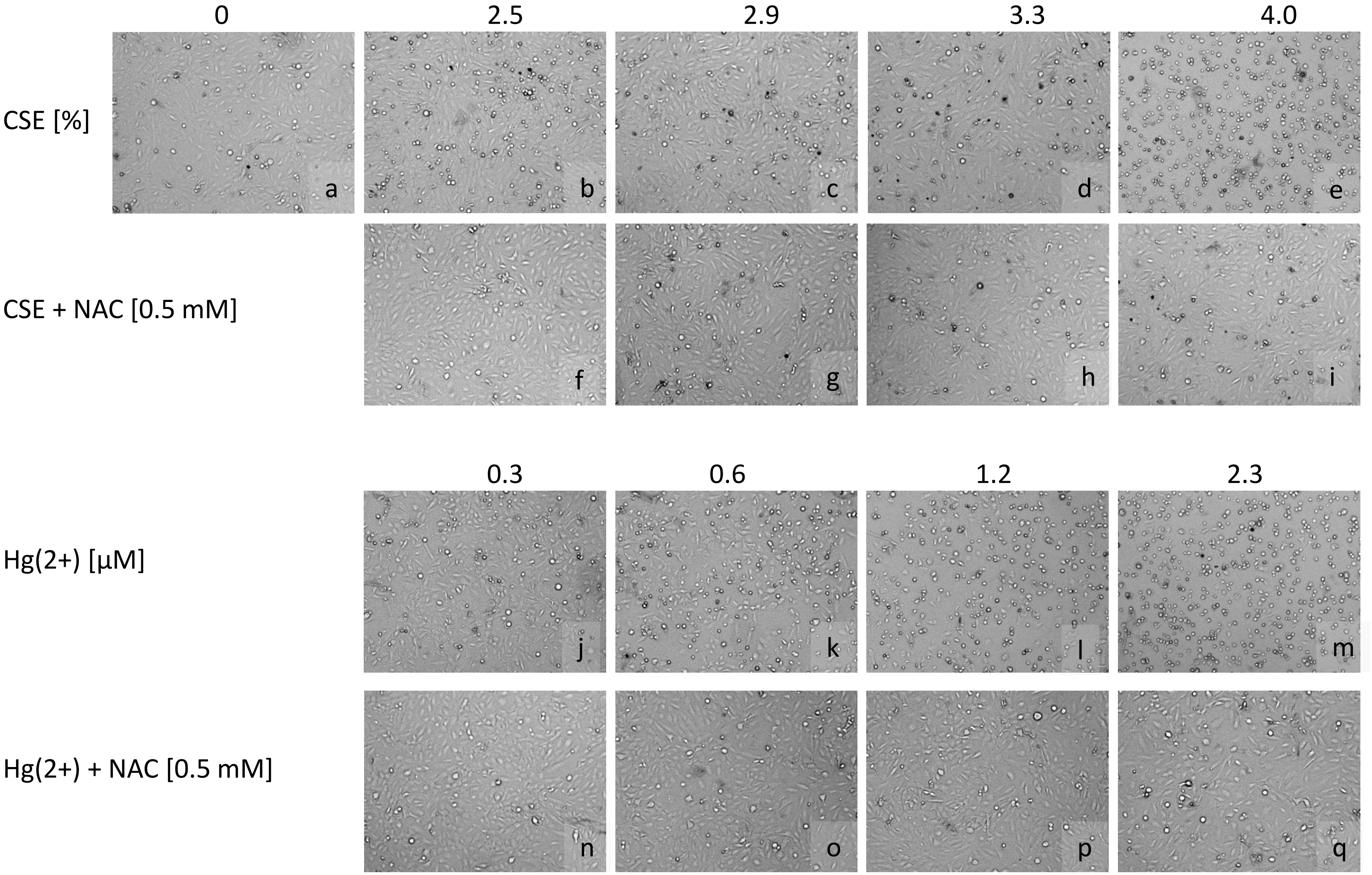

Supplementary Figure S3

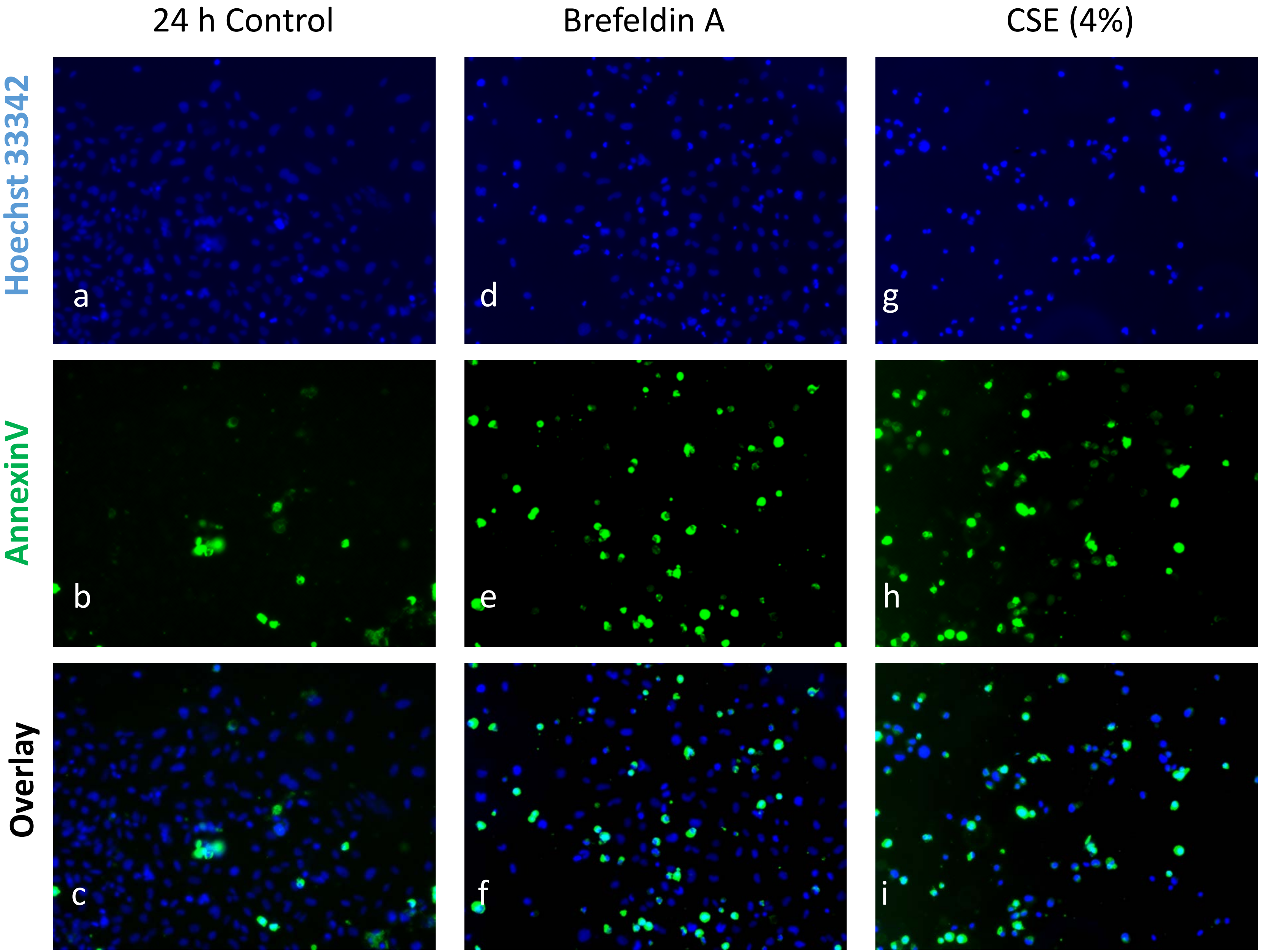

Supplementary Figure S4

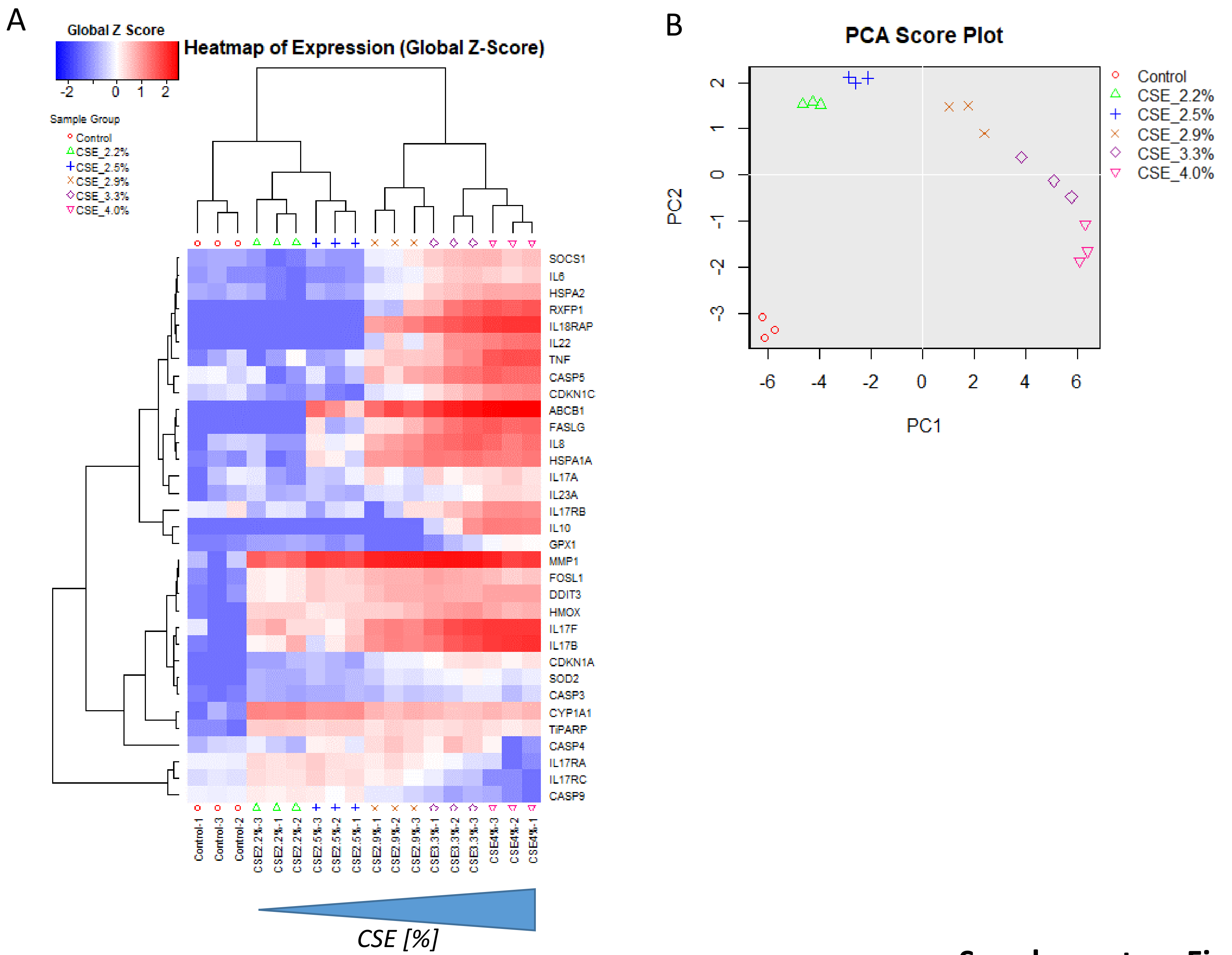

Supplementary Figure S5

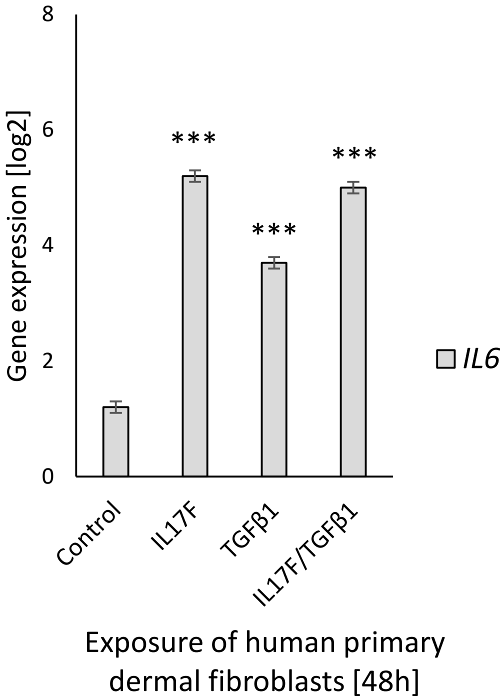

Supplementary Figure S6

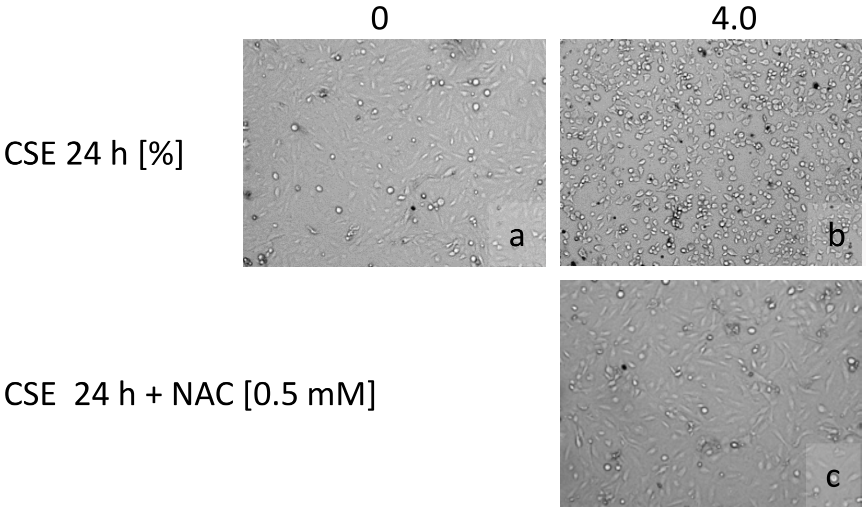

Supplementary Figure S7

Supplementary Table S1: Primer sequences and universal probe library probes (UPL) used for qPCR

Gene	Primer_forward	Primer_reverse	UPL	Remarks
ABCB1	ccgtggggcaagtcarttcatt	ccggtcgggtgggatagttga	66	
ACTA2	gctgttttcccatccattgt	agggtgggatgctcttcag	w/o	fibrosis
CASP3	ctggttttcggtgggtgt	ccactgagttttcagtgttctcc	34	
CASP4	ttcctggcaattgaaaatgg	tgcaagctgtactaatgaaggtg	23	
CASP5	cctctttcctggcaattgaa	tgctctttgatgttgacagagg	27	
CASP9	cccaagctcttttcatcca	ttactgccaggggactcgt	32	
CDKN1A	ccgaggcactcagaggag	agctgctcgctgtccact	70	
CDKN1C	ctcctttccccttcttctcg	tccatcgtggatgtgctg	55	
COL1A1	gtcgcactggtgatgctg	ggtggtgtccacctcgag	w/o	fibrosis
CYP1A1	tccaagagtccacccttcc	aagcatgatcagtgtagggatct	66	
DDIT3	aaggcactgagcgtatcatgt	tgaagatacacttccttcttgaacac	87	
FASLG	tggggatgtttcagctcttc	gtgtgcatctggctggtaga	17	
FN1	ccagtccacagctattcctg	acaaccacggatgagctg	w/o	isoform: ED-A FN1; fibrosis
FOSL1	aaccggaggaaggaactgac	ctgcagcccagatttctca	4	
GAPD	gctctctgctcctcctgttc	acgaccaaatccgttgactc	60	reference gene
GAPD	gagtcaacggatttggtcgt	ttgattttggagggatctcg	w/o	reference gene
GPX1	caaccagtttgggcatcag	gttcacctcgcacttctcg	77	
GUSB	cgccctgcctatctgtattc	tccccacagggagtgtgtag	57	reference gene
HMOX	cagtcaggcagagggtgatag	cctgcaactcctcaaagagc	15	
HSPA1A	ggagtcctacgccttcaaca	ccagcaccttcttcttgtcg	88	
HSPA2	cgaggttaccttcgacattga	tgttttccttaccggtgctc	70	
IL10	gatgccttcagcagagtgaa	gcaacccaggtaacccttaaa	67	
IL17A	ccccaaagcagttagactatgg	ttgaaggatgagggtcctg	23	
IL17B	gtggatgtccaacaagaggag	ctggggtcgtggttgatg	45	
IL17C	ccctcagctacgacccagt	cttctgtggatagcggtcct	16	
IL17D	gccctgggcctacagaat	acgacggtgggcatgtag	27	
IL17E	catgtaccaggtcagtgcaga	tctgctcctctccctctcag	27	
IL17F	ggcatcatcaatgaaaacca	tggggtcccaagtgacag	10	
IL17RA	gtcatcctgctcatcgtctg	tggtgtcatcactgtatttttcact	85	
IL17RB	tgtcgctcgtgctgctaa	actctggagatggcccagt	21	
IL17RC	cgaagtctggaacagcatcc	cagatgcacgttgtcaccat	3	
IL18RAP	cagatattctggatcctgtcgag	tgctttgcagctaatagttaaagg	47	
IL22	caacaggctaagcacatgtca	actgtgtccttcagcttttgc	6	
IL23A	cagcttcatgcctccctact	gactgaggcttggaatctgc	14	
IL6	gatgagtacaaaagtcctgatcca	ctgcagccactggttctgt	40	
IL6	ccccagtacccccaggagaagat	gctgagatgccgtcgaggatgta	w/o	
IL8	agacagcagagcacacaagc	aggaaggctgccaagagag	72	
MMP1	acgaatttgccgacagagat	gtccttggggtatccgtgta	26	
MUC5AC	agcaccagtgcccaagtct	actcctggcagtccatgc	43	
PALLD	ttgaaggtgccagttcaaca	tcaggtcggcagagatatga	w/o	fibrosis
PGK1	ctgtgggggtatttgaatgg	cttccaggagctccaaactg	w/o	reference gene
PPIA	ttcatctgcactgccaagac	tcgagttgtccacagtcagc	w/o	reference gene
RORC	cagcgctccaacatcttct	ccacatctcccacatggact	69	
RXFP1	tggagtatgcttccctcttca	tgcggccaaattaatacca	59	
SOCS1	ccccctggttgttgtagcag	gtaggaggtgcgagttcagg	36	
SOCS3	gacttcgattcgggaccag	aacttgctgtgggtgaccat	70	
SOD2	ctggacaaacctcagcccta	tgatggcttccagcaactc	77	
TiPARP	ggaaattcttctgtagggacca	gttggcttcttcaatcaatcg	58	
TNF	cagcctcttctccttcctgat	gccagagggctgattagaga	29	

Supplementary Table S2. Change in gene expression (mean, x-fold) compared to control after 24 h of exposure.
highlighted are values with p<0.0013 (Bonferroni correction)

Cell type		BEAS										THP-1								PBMC	
Exposure		Aqueous cigarette smoke extract (CSE) [%]																			
		2.2		2.5		2.9		3.3		4.0		2.2		2.9		4.0		5.0		4.0	
Gene	Function	x-fold	*p-value*	x-fold	*p-value*	x-fold	*p-value*	x-fold	*p-value*	x-fold	*p-value*	x-fold	*p-value*	x-fold	*p-value*	x-fold	*p-value*	x-fold	*p-value*	x-fold	*p-value*
RXFP1	adhesion, relaxin receptor	1.0	*0.9616*	1.0	*0.6854*	1.2	*0.8748*	18.1	*0.0494*	**83.1**	*0.0012*	-3.8	*0.0345*	-21.7	*0.0030*	-2.3	*0.0082*	-1.9	*0.0082*	n.d.	
CASP3	apoptosis	1.3	*0.0020*	1.4	*0.0026*	1.7	*0.0021*	**2.0**	*<0.001*	1.9	*0.0072*	1.4	*0.0387*	1.6	*0.0081*	1.8	*0.0237*	1.1	*0.4855*	1.8	*0.0138*
CASP4	apoptosis	1.0	*0.8182*	1.9	*0.0389*	3.5	*0.0017*	3.4	*0.0152*	-1.2	*0.7507*	1.6	*0.3637*	3.6	*0.0032*	-1.5	*0.0809*	-1.7	*0.0179*	**-3.8**	*0.0011*
CASP5	apoptosis	-1.7	*0.2078*	-1.5	*0.1899*	4.0	*0.0124*	22.1	*0.0063*	**49.3**	*<0.001*	2.4	*0.0191*	17.1	*0.0043*	41.0	*0.0012*	5.5	*0.0220*	-1.7	*0.0108*
CASP9	apoptosis	**1.5**	*<0.001*	1.5	*0.0140*	-1.2	*0.0401*	**-1.8**	*0.0010*	**-2.4**	*<0.001*	1.4	*0.0442*	3.0	*0.0082*	-1.8	*0.0201*	-1.3	*0.1159*	1.3	*0.0223*
FASLG	apoptosis	1.1	*0.8468*	12.6	*0.0035*	**36.3**	*<0.001*	**372.4**	*<0.001*	**807.1**	*<0.001*	1.0	*0.8091*	1.0	*0.7071*	**4.6**	*<0.001*	**154.0**	*<0.001*	**-4.8**	*0.0010*
FOSL1	apoptosis	**3.9**	*<0.001*	**6.4**	*<0.001*	**11.6**	*<0.001*	**14.5**	*<0.001*	**8.0**	*<0.001*	2.4	*0.0290*	10.6	*0.0053*	5.1	*0.0141*	3.4	*0.0018*	**4.0**	*<0.001*
TiPARP	cellular stress, DNA damage	**6.7**	*<0.001*	**6.2**	*<0.001*	**5.4**	*<0.001*	**5.3**	*<0.001*	**4.5**	*<0.001*	2.6	*0.0045*	**8.2**	*<0.001*	**5.9**	*<0.001*	**4.6**	*<0.001*	1.9	*0.0158*
HSPA1A	cellulare stress	1.1	*0.8068*	3.0	*0.0229*	**25.3**	*<0.001*	**58.2**	*<0.001*	**47.8**	*<0.001*	2.0	*0.0016*	**17.2**	*<0.001*	**144.4**	*<0.001*	38.6	*0.0030*	**17.4**	*<0.001*
HSPA2	cellulare stress	-1.3	*0.1095*	-1.0	*0.8171*	2.2	*0.0049*	**6.4**	*<0.001*	**11.6**	*<0.001*	1.4	*0.0042*	3.2	*0.0038*	**16.4**	*<0.001*	**13.5**	*<0.001*	**15.5**	*<0.001*
ABCB1	drug metabolism	1.0	*0.3739*	7.7	*0.0674*	**61.6**	*<0.001*	**1212.8**	*<0.001*	**13809.1**	*<0.001*	-1.4	*0.4226*	7.1	*0.0226*	64.6	*0.0059*	63.7	*0.0061*	-2.5	*0.0056*
CYP1A1	drug metabolism, AhR-related	**37.7**	*<0.001*	**25.4**	*<0.001*	**10.1**	*<0.001*	**8.2**	*<0.001*	**9.3**	*<0.001*	9.6	*0.0071*	44.7	*0.0025*	1.7	*0.3108*	1.6	*0.2420*	**-151.2**	*<0.001*
IL17A	IL17 family	-1.1	*0.8896*	1.3	*0.4676*	2.1	*0.1510*	3.0	*0.0261*	3.5	*0.0141*	1.5	*0.2189*	6.4	*0.0622*	10.2	*0.0411*	9.8	*0.0081*	-1.4	*0.6898*
IL17B	IL17 family	6.0	*0.0440*	4.4	*0.0861*	**39.2**	*<0.001*	171.4	*0.0054*	**1043.1**	*<0.001*	1.8	*0.1487*	1.1	*0.8091*	**7.9**	*<0.001*	4.4	*0.0181*	**4.2**	*<0.001*
IL17C	IL17 family	1.4	*0.2349*	1.2	*0.6855*	2.3	*0.1215*	2.7	*0.0043*	-1.1	*0.8524*	2.3	*0.1856*	-1.2	*0.8793*	2.3	*0.7950*	1.7	*0.5188*	**-5.2**	*0.0011*
IL17D	IL17 family	-1.1	*0.7222*	-1.1	*0.8848*	1.2	*0.3880*	1.3	*0.2243*	1.1	*0.7007*	-1.1	*0.6610*	-2.7	*0.3362*	1.2	*0.9775*	-1.1	*0.9046*	1.1	*0.2050*
IL17E	IL17 family	1.1	*0.9646*	-1.2	*0.7165*	1.1	*0.4878*	1.0	*0.8253*	0.9	*0.6754*	-1.2	*0.8371*	1.0	*0.6400*	1.1	*0.5378*	1.1	*0.4502*	1.3	*0.1959*
IL17F	IL17 family	8.1	*0.0251*	6.4	*0.3416*	**42.2**	*<0.001*	**272.8**	*<0.001*	**769.4**	*<0.001*	5.4	*0.7329*	6.1	*0.4226*	**288.2**	*<0.001*	**301.3**	*<0.001*	**198.5**	*<0.001*
IL17RA	IL17 receptor family	**2.0**	*<0.001*	2.1	*0.0060*	1.7	*0.0117*	1.0	*0.7836*	-2.1	*0.0334*	1.5	*0.1109*	-1.2	*0.1947*	-1.8	*0.0363*	-1.1	*0.3037*	**-3.5**	*<0.001*
IL17RB	IL17 receptor family	-1.5	*0.1861*	-1.6	*0.1837*	-1.5	*0.5033*	2.8	*0.0519*	**10.7**	*<0.001*	2.2	*0.1829*	7.7	*0.0261*	43.7	*0.0076*	8.2	*0.0261*	-2.2	*0.0069*
IL17RC	IL17 receptor family	**2.3**	*<0.001*	**2.5**	*0.0011*	1.7	*0.0052*	-1.1	*0.4969*	**-2.1**	*<0.001*	3.1	*<0.001*	2.9	*0.0399*	-1.4	*0.5778*	1.0	*0.8845*	3.3	*0.0015*
IL23A	IL17-related	1.3	*0.2480*	1.2	*0.2652*	1.7	*0.0230*	2.2	*0.0098*	**4.4**	*<0.001*	1.2	*0.0423*	-1.1	*0.2979*	1.4	*0.2021*	1.9	*0.0503*	1.2	*0.2451*
RORC	IL17-related	**-3.1**	*<0.001*	-9.8	*0.0290*	**-46.2**	*0.0013*	**-16.9**	*<0.001*	-22.0	*0.0135*	-1.4	*0.6345*	-1.0	*0.8648*	-1.2	*0.3748*	-1.4	*0.5748*	1.1	*0.9073*
IL10	inflammation	1.0	*0.8838*	1.0	*0.2283*	1.0	*0.1819*	6.9	*0.1589*	**85.5**	*<0.001*	-1.8	*0.0042*	-2.7	*0.0078*	**-3.9**	*0.0011*	**-10.4**	*<0.001*	**-6.8**	*<0.001*
IL18RAP	inflammation	1.1	*0.8446*	-1.2	*0.7546*	**15.7**	*<0.001*	**159.0**	*<0.001*	**674.8**	*<0.001*	-1.2	*0.3915*	-1.3	*0.4615*	-1.2	*0.8053*	-2.5	*0.8233*	1.3	*0.2627*
IL22	inflammation	3.7	*0.0037*	4.2	*0.1220*	2.4	*0.1931*	16.1	*0.0190*	**52.1**	*<0.001*	1.1	*0.3314*	1.0	*0.8091*	5.4	*0.0098*	**61.0**	*<0.001*	**22.7**	*<0.001*
IL6	inflammation	-1.2	*0.1164*	-1.0	*0.4350*	1.9	*0.0132*	**5.8**	*<0.001*	**5.4**	*<0.001*	1.0	*0.3911*	2.7	*0.0555*	2.9	*0.0286*	1.0	*0.8092*	**-4.2**	*<0.001*
IL8	inflammation	-1.1	*0.7886*	2.3	*0.0343*	13.0	*0.0018*	**77.4**	*<0.001*	**53.4**	*<0.001*	**5.6**	*<0.001*	**33.6**	*<0.001*	6.1	*0.0220*	5.1	*0.0018*	**-1.5**	*<0.001*
TNF	inflammation	1.1	*0.7759*	1.2	*0.6354*	5.0	*0.0028*	14.7	*0.0059*	**217.8**	*<0.001*	4.0	*0.0043*	4.6	*0.0019*	2.7	*0.0368*	3.4	*0.0196*	1.6	*0.0058*
DDIT3	marker of ER stress	**3.8**	*<0.001*	**5.8**	*<0.001*	**11.2**	*<0.001*	**12.9**	*<0.001*	**16.9**	*<0.001*	**3.7**	*<0.001*	**7.6**	*<0.001*	**4.7**	*<0.001*	**5.4**	*<0.001*	**5.9**	*<0.001*
MUC5AC	mucosa protection, IL17-related	1.4	*0.1527*	-3.7	*0.1697*	-6.0	*0.0142*	2.9	*0.2775*	**15.9**	*<0.001*	n.d.		n.d.		n.d.		n.d.		n.d.	
CDKN1C	negative regulator of cell proliferation	-1.7	*0.0082*	-1.8	*0.0083*	1.3	*0.0897*	3.9	*0.0053*	**14.3**	*<0.001*	8.2	*0.0025*	**22.1**	*<0.001*	**54.5**	*<0.001*	**51.4**	*<0.001*	**7.4**	*<0.001*
SOD2	oxidative stress	**1.4**	*<0.001*	**1.6**	*<0.001*	**2.1**	*<0.001*	**3.0**	*<0.001*	**2.6**	*<0.001*	1.7	*0.0454*	2.8	*0.0031*	5.0	*<0.001*	3.1	*0.0030*	**-2.3**	*<0.001*
HMOX	oxidative stress, NRF2-related	**6.8**	*<0.001*	5.5	*<0.001*	**8.0**	*<0.001*	**21.6**	*<0.001*	**13.8**	*<0.001*	**157.2**	*<0.001*	**1855.4**	*<0.001*	**424.2**	*<0.001*	**32.7**	*0.0021*	1.2	*0.0533*
GPX1	peroxidase	1.2	*0.0036*	1.1	*0.0520*	-1.1	*0.0118*	1.4	*0.1027*	**3.1**	*<0.001*	1.1	*0.3314*	-2.0	*0.0212*	1.6	*0.0216*	2.2	*0.0111*	**-2.6**	*<0.001*
CDKN1A	senescence	1.4	*0.0051*	1.5	*0.0116*	**2.6**	*<0.001*	**4.6**	*<0.001*	**4.5**	*<0.001*	3.1	*0.0036*	**5.8**	*<0.001*	**4.9**	*<0.001*	**2.5**	*<0.001*	**2.4**	*<0.001*
SOCS1	suppressor of cytokine signaling	-1.2	*0.0369*	-1.1	*0.1766*	2.3	*0.0074*	**6.9**	*<0.001*	**7.1**	*<0.001*	**7.4**	*0.0011*	**25.2**	*<0.001*	**35.0**	*<0.001*	**27.2**	*<0.001*	-1.4	*0.0130*
SOCS3	SOCS, IL-17-related	1.2	*0.3953*	1.0	*0.9446*	-1.9	*0.2742*	1.7	*0.0969*	1.9	*0.0259*	4.2	*0.0074*	2.3	*0.6923*	10.5	*0.0261*	3.9	*0.0837*	**4.2**	*<0.001*
MMP1	tissue remodeling, collagenase	**89.8**	*<0.001*	**503.8**	*<0.001*	**3727.9**	*<0.001*	**9200.5**	*<0.001*	**1077.4**	*<0.001*	9.5	*0.0903*	10.9	*0.0532*	**12.2**	*0.0013*	**4.7**	*<0.001*	**-21.1**	*<0.001*

n.d.; not detectable

Cell type		BEAS													
Exposure		Mercury [µM]								Cadmium [µM]				Brefeldin A [µM]	
		0.29		0.58		1.2		2.3		19		38		17.8	
Gene	Function	x-fold	*p-value*	x-fold	*p-value*	x-fold	*p-value*	x-fold	*p-value*	x-fold	*p-value*	x-fold	*p-value*	x-fold	*p-value*
RXFP1	adhesion, relaxin receptor	1.0	*0.3150*	1.0	*0.1128*	9.9	***<0.001***	42.4	*0.0287*	1.0	*0.6410*	**118.2**	*<0.001*	1.1	*0.8856*
CASP3	apoptosis	-1.3	*0.0076*	-1.3	*0.0399*	-1.1	*0.4378*	-1.3	*0.0190*	**-1.5**	*<0.001*	**-5.8**	*<0.001*	2.5	*0.0044*
CASP4	apoptosis	-1.0	*0.9863*	4.3	*0.0374*	1.8	*0.4033*	2.1	*0.3617*	-5.7	*0.0254*	1.2	*0.7278*	-1.1	*0.9654*
CASP5	apoptosis	1.1	*0.8088*	1.8	*0.1504*	8.1	*0.0093*	**33.5**	*<0.001*	1.3	*0.5748*	**40.7**	*<0.001*	1.1	*0.8946*
CASP9	apoptosis	**-1.4**	*<0.001*	**-2.4**	*<0.001*	**-3.1**	*<0.001*	**-3.7**	*<0.001*	**-1.6**	*<0.001*	**-2.0**	*<0.001*	2.0	*0.0028*
FASLG	apoptosis	1.0	*0.3092*	3.2	*0.0101*	**8.2**	*<0.001*	**50.7**	*<0.001*	1.0	*0.6521*	**17.1**	*<0.001*	-1.1	*0.9854*
FOSL1	apoptosis	1.8	*0.0011*	**4.2**	*<0.001*	**7.6**	*<0.001*	**5.4**	*<0.001*	3.0	*<0.001*	**15.0**	*<0.001*	3.2	*0.0598*
TiPARP	cellular stress, DNA damage	-1.0	*0.9820*	1.4	*0.0142*	**2.5**	*<0.001*	**3.3**	*<0.001*	1.2	*0.2252*	**3.2**	*<0.001*	2.4	*0.2065*
HSPA1A	cellulare stress	-1.4	*0.0076*	**19.9**	*<0.001*	**44.4**	*<0.001*	**101.0**	*<0.001*	**4.4**	*<0.001*	**134.3**	*<0.001*	-2.7	*0.0506*
HSPA2	cellulare stress	-1.7	*<0.001*	1.4	*0.0385*	**3.4**	*<0.001*	**10.5**	*<0.001*	-1.2	*0.0370*	**1.6**	*<0.001*	-3.5	*0.0483*
ABCB1	drug metabolism	-1.0	*0.1778*	**8.5**	*<0.001*	**37.2**	*<0.001*	**619.9**	*<0.001*	3.1	*0.0417*	**1791.1**	*<0.001*	1.0	*0.8654*
CYP1A1	drug metabolism, AhR-related	-1.4	*0.2777*	-1.3	*0.3150*	1.2	*0.3502*	1.7	*0.1747*	1.2	*0.6300*	**5.2**	*<0.001*	1.6	*0.3100*
IL17A	IL17 family	1.1	*0.3042*	3.1	*0.0897*	**1.6**	*<0.001*	6.2	*0.0039*	-1.6	*0.0211*	**4.2**	*<0.001*	-2.2	*0.4599*
IL17B	IL17 family	-4.1	*0.2307*	1.2	*0.5829*	6.5	*0.0087*	**80.8**	*<0.001*	1.3	*0.5405*	**103.5**	*<0.001*	6.1	*0.0748*
IL17C	IL17 family	-2.5	*0.1471*	1.3	*0.6835*	-2.2	*0.2333*	-1.1	*0.8580*	-1.3	*0.6571*	-1.6	*1.0000*	-2.5	*0.3264*
IL17D	IL17 family	-2.1	*0.0030*	**-4.3**	*<0.001*	-2.5	*0.0039*	-1.7	*0.0352*	-1.5	*0.0957*	-1.2	*0.3854*	4.9	*0.0107*
IL17E	IL17 family	-1.3	*0.3483*	-1.1	*0.2844*	1.5	*0.5317*	1.1	*0.6270*	1.2	*0.4423*	-1.1	*0.2979*	1.1	*0.8246*
IL17F	IL17 family	2.3	*0.2817*	2.4	*0.1188*	4.0	*0.1040*	**38.5**	*<0.001*	1.0	*1.0000*	**32.0**	*<0.001*	14.9	*0.0124*
IL17RA	IL17 receptor family	1.1	*0.3091*	-1.0	*0.9317*	-1.5	*0.0245*	**-2.3**	*<0.001*	-1.3	*0.0927*	-1.7	*0.0069*	2.3	*0.0078*
IL17RB	IL17 receptor family	-2.3	*0.0071*	-2.5	*0.0051*	1.3	*0.5581*	**6.7**	*<0.001*	-1.6	*0.1211*	-1.3	*0.3476*	**3.6**	*<0.001*
IL17RC	IL17 receptor family	1.2	*0.0261*	-1.3	*0.0037*	-1.9	*0.0011*	**-4.9**	*<0.001*	-1.2	*0.0433*	**-2.1**	*<0.001*	3.7	*0.0036*
IL23A	IL17-related	-1.1	*0.5298*	1.6	*0.3032*	1.1	*0.6200*	**5.0**	*<0.001*	1.2	*0.1997*	**10.7**	*<0.001*	1.1	*0.8846*
RORC	IL17-related	-3.7	*0.4175*	-6.7	*0.6251*	-5.4	*0.3725*	-15.9	*0.2740*	-3.3	*0.0626*	-10.5	*0.1460*	-1.1	*0.3858*
IL10	inflammation	1.0	*0.9317*	1.0	*0.5581*	2.2	*0.0328*	**25.6**	*<0.001*	1.0	*0.2258*	**139.7**	*<0.001*	1.0	*0.6200*
IL18RAP	inflammation	1.0	*1.0000*	1.8	*0.0047*	**20.1**	*<0.001*	**343.9**	*<0.001*	**21.0**	*<0.001*	**2290.8**	*<0.001*	1.0	*1.0000*
IL22	inflammation	1.0	*0.3715*	-1.9	*0.3896*	2.6	*0.1299*	3.6	*0.0202*	1.0	*0.8977*	15.2	*0.0017*	1.0	*1.0000*
IL6	inflammation	-1.3	*0.3433*	-1.4	*0.2444*	1.5	*0.1935*	**7.9**	*<0.001*	1.7	*0.1075*	**8.8**	*<0.001*	5.0	*0.0631*
IL8	inflammation	2.0	*<0.001*	**6.4**	*<0.001*	**14.2**	*<0.001*	**53.7**	*<0.001*	**4.3**	*<0.001*	**133.6**	*<0.001*	-1.8	*0.5608*
TNF	inflammation	-1.4	*0.2795*	-1.1	*0.7895*	2.5	*0.0274*	**13.1**	*<0.001*	-1.0	*0.9472*	**58.5**	*<0.001*	-1.7	*0.4784*
DDIT3	marker of ER stress	1.1	*0.2167*	**3.4**	*<0.001*	**7.1**	*<0.001*	**12.1**	*<0.001*	**1.8**	*<0.001*	**17.5**	*<0.001*	**40.5**	*<0.001*
MUC5AC	mucosa protection, IL17-related	-6.6	*0.1016*	-1.5	*0.3155*	**5.0**	*<0.001*	**45.1**	*<0.001*	2.8	*0.0033*	**260.8**	*<0.001*	-5.2	*0.4668*
CDKN1C	negative regulator of cell proliferation	-2.7	*<0.001*	2.0	*0.0013*	**8.0**	*<0.001*	**20.8**	*<0.001*	**2.7**	*<0.001*	**189.3**	*<0.001*	-1.5	*0.5387*
SOD2	oxidative stress	-1.3	*0.0554*	1.2	*0.1209*	**2.0**	*<0.001*	**3.1**	*<0.001*	-1.0	*0.8987*	-1.4	*0.0033*	-1.0	*0.9972*
HMOX	oxidative stress, NRF2-related	**6.5**	*<0.001*	**29.2**	*<0.001*	**44.0**	*<0.001*	**56.6**	*<0.001*	**29.2**	*<0.001*	**244.2**	*<0.001*	3.4	*0.0019*
GPX1	peroxidase	**-3.6**	*<0.001*	**-7.0**	*<0.001*	**-7.0**	*<0.001*	**-4.1**	*<0.001*	**-3.4**	*<0.001*	**-1.8**	*<0.001*	-1.4	*0.5633*
CDKN1A	senescence	**-1.5**	*<0.001*	**1.4**	*<0.001*	**3.9**	*<0.001*	**6.0**	*<0.001*	**-1.7**	*<0.001*	**1.8**	*<0.001*	3.1	*0.0161*
SOCS1	suppressor of cytokine signaling	**-2.8**	*<0.001*	-1.1	*0.7065*	**2.0**	*<0.001*	**5.9**	*<0.001*	-1.1	*0.0252*	**10.3**	*<0.001*	-3.5	*0.1888*
SOCS3	SOCS, IL-17-related	-2.0	*0.0128*	-5.1	*0.0348*	-2.3	*0.0067*	-1.5	*0.2750*	-1.7	*0.0446*	-1.2	*0.5075*	-1.5	*0.7428*
MMP1	tissue remodeling, collagenase	**10.4**	*<0.001*	**170.7**	*<0.001*	**814.2**	*<0.001*	**1834.1**	*<0.001*	**53.5**	*<0.001*	**494.3**	*<0.001*	6.2	*0.0288*

n.d.; not detectable
